# MicroRNA-181a protects against pericyte apoptosis via directly targeting FOXO1: implication for ameliorated cognitive deficits in APP/PS1 mice

**DOI:** 10.18632/aging.102171

**Published:** 2019-08-29

**Authors:** Qingbin Wu, Xiaochen Yuan, Jing Bai, Ruiqin Han, Zhigang Li, Honggang Zhang, Ruijuan Xiu

**Affiliations:** 1 Key Laboratory for Microcirculation, Ministry of Health, Institute of Microcirculation, Chinese Academy Medical Sciences and Pecking Union Medical College, Beijing, China; 2 Department of Pharmacy, the Fourth Hospital of Hebei Medical University, Shijiazhuang, China; 3 Institute of Basic Medical Sciences, Chinese Academy of Medical Sciences, School of Basic Medicine Peking Union Medical College, Beijing, China; 4 School of Acupuncture, Moxibustion and Tuina, Beijing University of Chinese Medicine, Beijing, China

**Keywords:** Alzheimer’s disease, MicroRNA-181a, pericyte, FOXO1, apoptosis

## Abstract

MicroRNAs (miRNAs) have emerged as critical regulators in the pathology of Alzheimer’s disease (AD). MiR-181a is associated with hippocampal memory formation and aberrantly expressed in patients with mild cognitive impairment (MCI), however, little is known about its role and underlying mechanism involved in AD. Here, we report that miR-181a expression declines in APP/PS1 mice, synchronous with the increase in amyloid β (Aβ) level, which suggests a reverse correlation between miR-181a level and AD development. Additionally, lentiviral overexpression of miR-181a via intrahippocampal injection ameliorates cognitive deficits and amyloid plaque deposition in APP/PS1 mice, indicating a beneficial role of miR-181a against AD progression. Moreover, miR-181a decelerates pericyte loss and blood-brain barrier breakdown in APP/PS1 mice. Furthermore, miR-181a protects against Aβ accumulation-induced pericyte apoptosis *in vitro*, which is attributed to the negative regulation of FOXO1 by miR-181a, since FOXO1 restoration abolishes miR-181a protective role against pericyte apoptosis. Altogether, these results may identify miR-181a as a novel regulator of AD pathology, and also implicate that the protection of miR-181a in blood-brain barrier pericytes may underlie its ameliorating effect on APP/PS1 mice.

## INTRODUCTION

Pericytes are a type of vascular mural cells typically localized in the basement membrane of blood microvessels [1]. In the central nervous system (CNS), pericytes are distributed in the blood-brain barrier (BBB), where they are centrally positioned in the neurovascular unit (NVU), which is comprised of endothelial cells, astrocytes and neurons [2]. Pericytes cooperate with neighboring cells through complicated signaling pathways to ensure key and normal CNS functions, such as BBB integrity maintenance, vascular stability, and toxic byproduct clearance [3–5]. Pericyte degeneration and BBB breakdown have been found in some neurological disorders, such as spinal cord injury [6], including Alzheimer’s disease (AD) [7, 8], a neurodegenerative disease characterized by abnormally elevated amyloid β-peptide (Aβ), tau pathology and neuronal loss, which lead to progressive cognitive decline, and ultimately, dementia [9, 10]. In transgenic APP/PS1 mice, progressive degeneration of pericytes was found to accelerate Alzheimer-like neurodegeneration [11], suggesting that counteracting pericyte loss may have therapeutic benefit in modifying AD progression.

MicroRNAs (miRNAs), a class of single-stranded and non-coding RNAs with 18–22 nucleotides, play versatile biological functions through post-transcriptional regulation of gene expression via targeting the 3’-untranslated region (3’UTR) of target mRNAs [12]. Escalating evidence has suggested that several miRNAs not only have a great potential as biomarkers in AD diagnosis [13–15], but also are mechanistically associated with AD pathology through distinct mechanisms, such as Aβ level regulation, Tau phosphorylation and synaptic damage, etc [13, 16]. However, despite of these, the potential contribution of miRNAs to AD pathology still requires further elucidation.

Recently, miR-181a has been implicated in hippocampus-dependent memory formation [17, 18] and the cognitive function of epileptic rats [19]. In plasma, aberrantly expressed miR-181a was also found in patients with mild cognitive impairment (MCI), an early stage of neurodegeneration frequently associated with AD [20, 21]. Yet, as far as we know, whether miR-181a is associated with AD has not been reported. In this study, through taking advantage of transgenic APP/PS1 mice, a murine AD model, we show an ameliorating effect of miR-181a on cognitive deficits, which may relate to the retarded pericyte loss and blood-brain barrier breakdown via a negative regulation of pericyte apoptosis. We propose that miR-181a functions as a negative regulator of AD progression, which may be exploited as a potential target in for AD treatment.

## RESULTS

### MiR-181a expression is downregulated in APP/PS1 mice during AD development

**Figure 1:**
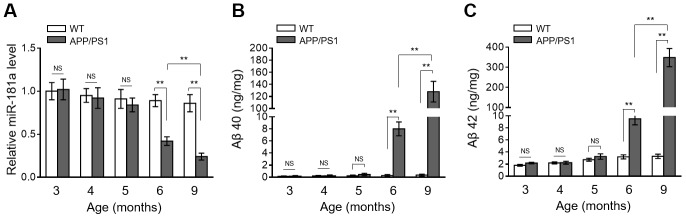
**MiR-181a expression declines in APP/PS1 mice during AD development.** (**A**) The expression of miR-181a in the brain of wild-type (WT) and APP/PS1 mice with increasing indicated age was determined by qRT-PCR analysis. U6 was used as an internal control. Data are expressed as relative to that of 3-month-old mice (n = 8 mice per group). (**B**–**C**) The level of Aβ 40 (**B**) and Aβ 42 (**C**) in WT and APP/PS1 mice with increasing indicated age was measured by ELISA assay. Data are expressed as ng Aβ 40 or Aβ 42 per mg total protein samples (n = 8 mice per group). All data are mean ± SD, and compared by one-way ANOVA followed by Tukey’s post-hoc tests. **, P < 0.01; NS, not significant.

To survey a possible connection between miR-181a and AD pathology, we first traced its expression pattern in the brain of APP/PS1 mice aged 3, 4, 5, 6 and 9 months old, a transgenic AD mice model manifesting disease symptoms with increasing age [22]. Analysis of qRT-PCR showed that the expression level of miR-181a started to display markedly decline in 6-month-old APP/PS1 mice, which became significantly lower in 9-month-old APP/PS1 mice, as compared with WT mice (Figure 1A). However, in 3-to-5 months old APP/PS1 mice, no significant change was observed in brain miR-181a expression level during this timeframe (Figure 1A). Meanwhile, astonishingly, in these APP/PS1 mice, the levels of Aβ 40 (Figure 1B) and Aβ 42 (Figure 1C) also showed significant upregulation beginning at 6 months of age, which were further drastically increased in 9-month-old APP/PS1 mice, in comparison with WT mice. The synchronous change of miR-181a downregulation and Aβ production in APP/PS1 mice suggests that a negative correlation may exist between miR-181a level and AD development.

### MiR-181a ameliorates cognitive deficits in APP/PS1 mice

**Figure 2:**
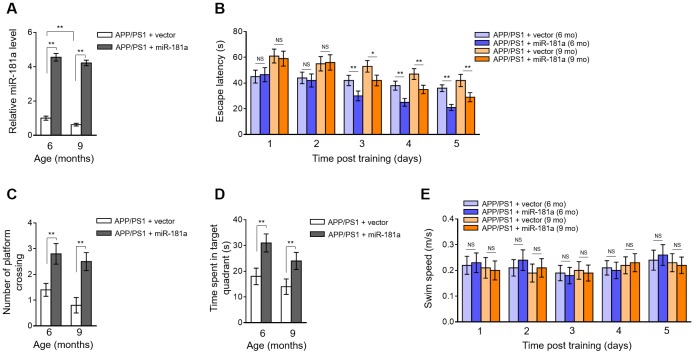
**Lentiviral overexpression of miR-181a via intrahippocampal injection ameliorates cognitive deficits in APP/PS1 mice**. (**A**–**E**) Lentiviral empty vector or lentiviral miR-181a expressing vector was injected into the hippocampus of APP/PS1 mice aged 5-month-old or 8-month-old. Eight mice were included in each group. One month later, mice were used for subsequent biochemical and behavior analyses. (**A**) The expression of miR-181a in the brain was determined by qRT-PCR analysis. U6 was used as an internal control. Data are expressed as relative to that of 6-month-old mice infected with lentiviral empty vector. (**B**) Spatial learning of APP/PS1 mice aged 6-month-old or 9-month-old was measured as escape latency (s) in water maze at different days after training. (**C**–**D**) Spatial memory of APP/PS1 mice was evaluated by probe trials performed at 24 h following the last training session. The number of platform location crossings (**C**) and time (s) spent in target quadrant (**D**) was recorded. (**E**) Swimming speed of APP/PS1 mice aged 6-month-old or 9-month-old was recorded. All data are mean ± SD, and compared by one-way ANOVA followed by Tukey’s post-hoc tests. **, P < 0.01; *, P < 0.05; NS, not significant.

We then asked whether miR-181a affects cognitive deficits in APP/PS1 mice. We addressed this issue by reversing its downregulation in the brain of 6-month-old and 9-month-old APP/PS1 mice through intrahippocampal injection of lentiviral vector expressing miR-181a [58]. The enforced overexpression of miR-181a in the brain of APP/PS1 mice was confirmed by qRT-PCR analysis (Figure 2A). We next assessed whether miR-181a affects spatial learning and memory in APP/PS1 mice via performing the Morris water maze test [23]. The results of escape latency in water maze after training showed that compared with vector group, miR-181a overexpression significantly improved learning and memory in those APP/PS1 mice (Figure 2B). In addition, as shown by the probe trials 24 h after the last training session, the number of platform location crossing (Figure 2C) as well as the time spent in the target quadrant (Figure 2D) were also decreased in APP/PS1 mice overexpressed with miR-181a. Noteworthily, no obvious changes were observed in swimming speed between two groups of mice (Figure 2E), indicating that the miR-181a-improved behavioral performances in APP/PS1 mice are attributed to cognitive processes, other than noncognitive behavioral activities. Altogether, these behavioral tests show that miR-181a overexpression in the brain ameliorates cognitive deficits in APP/PS1 mice, including impaired spatial learning and memory.

### MiR-181a ameliorates amyloid plaque deposition in APP/PS1 mice

**Figure 3:**
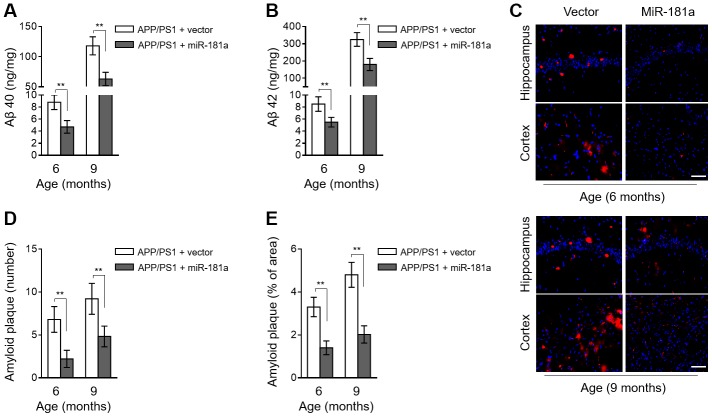
**MiR-181a ameliorates amyloid plaque deposition in APP/PS1 mice.** (**A**–**E**) Lentiviral empty vector or lentiviral miR-181a expressing vector was injected into the hippocampus of APP/PS1 mice aged 5-month-old or 8-month-old. Eight mice were included in each group. One month later, mice were used for subsequent biochemical analyses. (**A**–**B**) The level of Aβ 40 (**A**) and Aβ 42 (**B**) in APP/PS1 mice was measured by ELISA assay. Data are expressed as ng Aβ 40 or Aβ 42 per mg total protein samples. (**C**–**E**) The slices of mouse brain were stained with thioflavin-S to show plaques in hippocampus (upper) and cortex (lower). (**C**) The representative images are shown. The plaques were shown with red fluorescence and cell nuclei were highlighted by DAPI staining. Scale bar, 50 μm. (**D**–**E**) Quantification analysis of the number (**D**) and area (**E**) of amyloid plaque shown as in (**C**). All data are mean ± SD, and compared by one-way ANOVA followed by Tukey’s post-hoc tests. **, P < 0.01.

To further investigate the association between miR-181a and AD pathology, we checked whether miR-181a plays a role in amyloid plaque deposition, a hallmark pathologic change spotted in AD brain [24]. As analyzed by ELISA assay, compared with vector control, the levels of Aβ 40 (Figure 3A) and Aβ 42 (Figure 3B) were both decreased in APP/PS1 mice with miR-181a overexpression in the brain. To strengthen these results, we detected amyloid plaque in brain slices by Thioflavin-S staining. Consistently, we found that both the number (Figure 3C–3D) and area (Figure 3C and 3E) of amyloid plaque deposition in hippocampus and cortex of APP/PS1 mouse brain were markedly ameliorated by miR-181a overexpression. Thus, keeping in line with its alleviating effects on cognitive deficits in APP/PS1 mice (Figure 2), these results reveal that miR-181a reduces amyloid plaque deposition in APP/PS1 mice.

### MiR-181a decelerates pericyte loss and blood-brain barrier breakdown in APP/PS1 mice

**Figure 4:**
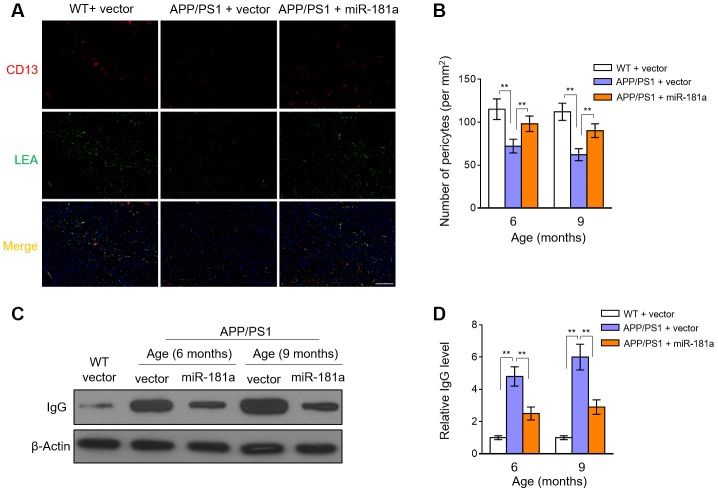
**MiR-181a decelerates pericyte loss and blood-brain barrier breakdown in APP/PS1 mice.** (**A**–**D**) Lentiviral empty vector or lentiviral miR-181a expressing vector was injected into the hippocampus of APP/PS1 mice aged 5-month-old or 8-month-old. Wild-type age-matched littermates were used as controls. Eight mice were included in each group. One month later, mice were used for subsequent biochemical analyses. (**A**) The representative immunofluorescent images of CD13-positive pericytes (red) and lectin-positive capillary endothelium (green) in 9-month-old WT and APP/PS1 mice. Scale bar, 100 μm. (**B**) Quantification of CD13-positive pericytes in the cortex and hippocampus of 6-month-old or 9-month-old WT and APP/PS1 mice. Results represent the number of CD13-positive pericytes per mm^2^. (**C**–**D**) The level of IgG in capillary-depleted cortical extracts from WT mice and 6-month-old or 9-month-old APP/PS1 mice was determined by Western blotting analysis. β-actin was used as a loading control. The representative blot images (**C**) and quantification analysis of IgG level (**D**) are shown. All data are mean ± SD, and compared by one-way ANOVA followed by Tukey’s post-hoc tests. **, P < 0.01.

MiRNAs, such as miR-149-5p, show the potential to regulate blood-brain barrier permeability through targeting pericytes [25]. It’s also well-known that pericyte loss and the ensuing disrupted integrity of BBB are inevitable consequences and also critical contributing factors to AD pathology [3, 26]. In accordance with previous studies [8, 11], the number of pericytes (CD13-positive) in the hippocampus and cortex was sharply decreased in 6-month-old and 9-month-old APP/PS1 mice compared with WT mice (Figure 4A–4B). Moreover, intriguingly, we noticed that in the brain of APP/PS1 mice, the decreased number of pericytes was significantly recovered by miR-181a overexpression (Figure 4A–4B). Therefore, these observations show that miR-181a retards pericyte loss in APP/PS1 mice. Pericyte loss-induced BBB breakdown leads to disrupted vascular permeability [27]. Keeping in line with the decelerated pericyte loss, miR-181a overexpression decreased the vascular leakage of IgG in APP/PS1 mice (Figure 4C–4D). Together, these data suggest that miR-181a protects against pericyte loss and vascular damage during AD development, offering an implication that the ameliorated cognitive deficits (Figure 2) and reduced amyloid plaque deposition (Figure 3) in APP/PS1 mice by miR-181a are associated with the protected pericytes and prevented BBB breakdown.

### MiR-181a prevents Aβ accumulation-induced pericyte apoptosis

**Figure 5:**
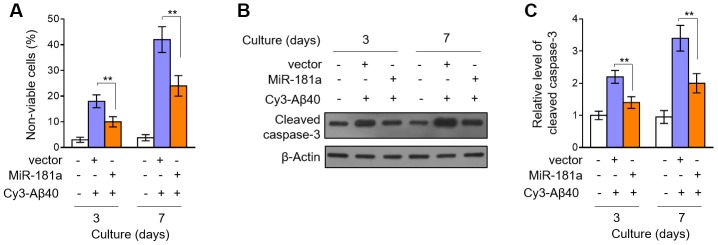
**MiR-181a protects against Aβ accumulation-induced pericyte apoptosis.** (**A**–**C**) Murine brain pericytes were isolated and cultured in DMEM medium. Pericytes were infected with lentiviral empty vector or lentiviral miR-181a expressing vector. Two days later, pericytes were cultured with or without 5 mM Aβ40 for consecutive 3 or 7 days. (**A**) Cell death was evaluated by Trypan blue staining. Results are expressed as percentage of Trypan blue positive cells (non-viable) among total cell number (%). Data are mean ± SD (n = 5). (**B**–**C**) The expression of cleaved caspase-3 was determined by Western blotting analysis. β-actin was used as a loading control. The representative blot images (**B**) and quantification analysis of cleaved caspase-3 expression (**C**) are shown. Data are mean ± SD (n = 3). Dara were compared by one-way ANOVA followed by Tukey’s post-hoc tests. **, P < 0.01; *, P < 0.05.

To learn more about the protective role of miR-181a in pericytes. We isolated the mouse pericytes from the microvessel fragments of mouse cortex and hippocampus, cultured them *in vitro* and then treated them with Aβ40, whose prolonged accumulation can cause cell death of pericytes [11]. Trypan blue exclusion assay showed that the increased number of non-viable cells by continuous 3 and 7 days of Aβ40 treatment was prominently attenuated when miR-181a was overexpressed in pericytes (Figure 5A). To assess whether miR-181a-prevented pericyte death is due to inhibited apoptosis, we next determined the expression of cleaved caspase-3, one typical marker of apoptosis induction [28]. Indeed, the results showed that the increased expression of cleaved caspase-3 in Aβ40-treated pericytes was suppressed by miR-181a overexpression (Figure 5B–5C), coinciding with reduced cell death upon Aβ40 treatment. Hence, these *in vitro* experiments illustrate that miR-181a inhibits Aβ40 accumulation-induced pericyte apoptosis, which is reminiscent of the attenuated pericyte loss in APP/PS1 mice overexpressing miR-181a.

### miR-181a protects against pericyte apoptosis by directly targeting FOXO1

**Figure 6:**
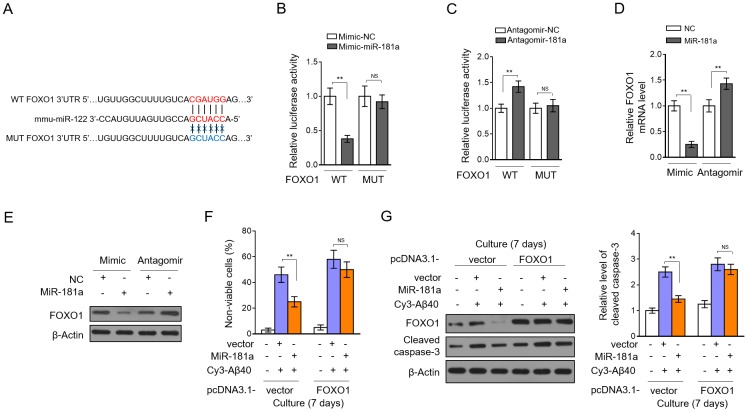
**miR-181a protects pericytes by directly targeting FOXO1.** (**A**) Specific binding sequence of miR-181a in the 3’-UTR of mouse FOXO1 mRNA (upper). The mutant sequence of 3’-UTR of mouse FOXO1 mRNA is also shown (below). (**B**) HEK293 cells were cotransfected negative control (NC) mimic or miR-181a mimic with dual-luciferase reporter plasmid expressing 3’-UTR of wild-type FOXO1 (WT) or mutant FOXO1 (MUT). Two days later, the luciferase activity was measured. Results are expressed as relative to NC. Data are mean ± SD (n = 3). (**C**) HEK293 cells were cotransfected negative control (NC) antagomir or miR-181a antagomir with dual-luciferase reporter plasmid expressing 3’-UTR of WT FOXO1 or MUT FOXO1. Two days later, the luciferase activity was measured. Results are expressed as relative to NC. Data are mean ± SD (n = 3). (**D**) Murine brain pericytes were transfected with NC mimic or miR-181a mimic, or NC antagomir or miR-181a antagomir. Three days later, the expression of FOXO1 was determined by qRT-PCR analysis. U6 was used as an internal control. Results are expressed as relative to NC. Data are mean ± SD (n = 3). (**E**) Pericytes were treated as in (**D**). The expression of FOXO1 was determined by Western blotting analysis. β-actin was used as a loading control. The representative blot images are shown (n = 3). (**F**–**G**) Murine brain pericytes were transfected with pcDNA3.1-vector or pcDNA3.1-FOXO1. Two days later, pericytes were cultured with or without 5 mM Aβ40 for consecutive 7 days. (**F**) Cell death was evaluated by Trypan blue staining. Results are expressed as percentage of Trypan blue positive cells (non-viable) among total cell number (%). Data are mean ± SD (n = 5). (**G**) The expression of FOXO1 and cleaved caspase-3 was determined by Western blotting analysis. β-actin was used as a loading control. The representative blot images (left) and quantification analysis of cleaved caspase-3 expression (right) are shown. Data are mean ± SD (n = 3). Dara were compared by one-way ANOVA followed by Tukey’s post-hoc tests. **, P < 0.01; NS, not significant.

To elucidate how miR-181a inhibits pericyte apoptosis under Aβ40 treatment, we predicted its potential targets through in silico TargetScan analysis [29]. We focused on the Forkhead transcription factor FKHR (FOXO1) (Figure 6A), since it was previously reported to play an essential role in pericyte apoptosis [30]. We first confirmed whether miR-181a directly targets the 3’UTR of FOXO1. As analyzed by luciferase reporter assay, in HEK293 cells, miR-181a overexpression substantially reduced the luciferase activity of wild-type 3’UTR of FOXO1, but meanwhile, did not have similar effect on that of the mutant construct (Figure 6B). In reverse, miR-181a knockdown significantly increased the luciferase activity of wild-type 3’UTR of FOXO1, however, with the mutant construct unaffected (Figure 6C). These results demonstrate that FOXO1 can be directly targeted by miR-181a. Further, we examined whether miR-181a regulates FOXO1 expression in pericytes. As shown in Figure 6D–6E, miR-181a overexpression reduced FOXO1 expression, and oppositely, its knockdown increased FOXO1 expression in pericytes, strengthening its negative role in regulating FOXO1 expression. We next asked whether the suppressed FOXO1 expression contributes to miR-181a protection against pericyte apoptosis induced by Aβ40 treatment. To test this hypothesis, we restored FOXO1 expression in pericytes through transient transfection-mediated overexpression. We found that miR-181a-rescued cell death (Figure 6F) and -attenuated pericyte apoptosis (Figure 6G) were completed abrogated along with FOXO1 restoration. Thus, these prove that the anti-apoptotic effect of miR-181a on Aβ40-treated pericytes relies on the targeted expression of FOXO1, uncovering the miR-181a/FOXO1 axis as an important regulator in inhibiting pericyte apoptosis, at least under the treatment of Aβ40 *in vitro*.

## DISCUSSION

**Figure 7:**
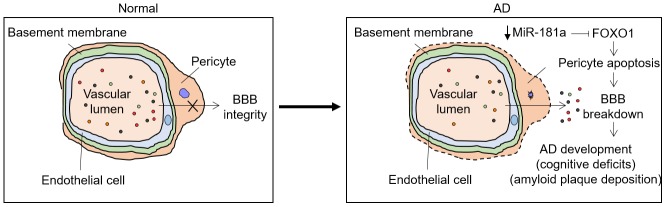
**Proposed model for this study.** MiR-181a is downregulated during AD development via an unknown mechanism. Normally, miR-181a targets and suppresses FOXO1, one positive regulator of pericyte apoptosis, which induces BBB breakdown and AD development, manifesting cognitive deficits and amyloid plaque deposition. Thus, this feedback regulation may accelerate AD development.

AD is a progressive neurodegenerative disorder and the leading cause of dementia in elder people. Although great advances have been achieved in understanding AD pathogenesis and great efforts have also been devoted to developing pharmacological therapeutics over the last two decades, current treatments, such as acetylcholinesterase inhibitors and N-methyl d-aspartate receptor antagonist, fail to generate satisfactory curative effect [10]. The altered expression profile of miRNAs in AD patients and its relevance to AD pathology have been recognized for many years, including the revealed significant role of some miRNAs in the regulation of both β-amyloid peptide and Tau toxicity [31–34]. Therefore, miRNAs hold promise to serve as prospective biomarkers and potential therapeutic targets for AD treatment. On the other hand, AD is characterized by various pathological changes in brain neurovascular system, such as degeneration of endothelial cells and pericytes, instability of vascular wall and disruption of BBB, etc, [35, 36]. Recently, the contribution of pericytes to AD pathology has increasingly gained particular interest [31]. In the present study, we discovered a previously unappreciated alleviating effect of miR-181a on AD progression, which may relate to its protective function against apoptosis-induced pericyte loss during AD development (Figure 7). The evidence supporting the above proposed notion is described as follows: 1) miR-181a expression declines in APP/PS1 mice synchronously with the accumulation of Aβ 40 and Aβ 42; 2) the overexpression of miR-181a via intrahippocampal injection of lentivirus ameliorates cognitive deficits in APP/PS1 mice; 3) miR-181a ameliorates amyloid plaque deposition in APP/PS1 mice; 4) miR-181a reduces pericyte loss and impedes BBB breakdown in APP/PS1 mice; 5) miR-181a protects against Aβ accumulation-induced pericyte apoptosis *in vitro*.

We noticed that miR-181a expression in the brain manifested significant decline in 6-month-old and 9-month-old APP/PS1 mice, which coincidently kept pace with the appearance of Aβ 40 and Aβ 42 upregulation. This result suggests that its downregulation is associated with AD pathology. Interestingly, miR-181a expression is significantly decreased in the serum and naïve CD4 T cells of elderly individuals [37, 38]. Although we did not find obvious decline in miR-181a expression in 9-month-old WT mice, presumably due to the short-term observation, it seems that its decline is accelerated in APP/PS1 mice, of which the underlying mechanisms are still unknown. It’s unlikely that the decreased miR-181a expression is associated with neuroinflammation in AD [39, 40], because its expression could be upregulated by inflammatory stimuli *in vivo* [41]. The decreased serum miR-181a has been proposed as a potential new tool for the diagnosis of breast cancer [42, 43]. Since there is an urgent need to identify non-invasive biomarkers for AD detection [44], it would be of clinical significance to investigate whether the serum miR-181a displays similar tendency in AD patients.

miR-181a overexpression in the brain rescues spatial learning and memory deficits and ameliorates amyloid plaque deposition in AD developing APP/PS1 mice, inversely suggesting that miR-181a decline along with AD development contributes to cognitive impairment and Aβ accumulation, and also implying that miR-181a may be considered as a therapeutic target for AD therapy. In previous studies, miR-181a selective overexpression or inhibition in the dorsal hippocampus (DH) enhances or impairs the hippocampus-dependent memory formation [17]. In addition, miR-181 silencing reduces injury and improves recovery in mice after focal cerebral ischemia [45, 46] and also exhibits neuroprotective role against hippocampus neuronal apoptosis in a rat model with epilepticus and in children with temporal lobe epilepsy [47]. However, it’s reported that the age-dependent decreased expression of miR-181 correlates with neuronal survival, and reversely, its overexpression increases the rate of apoptosis [48, 49]. These findings indicate that miR-181a plays a wide-spectrum of activities in CNS, and therefore manipulating its expression should be conducted with considerable cautiousness.

The pericyte degeneration leads to brain vascular damage, including BBB breakdown and brain microcirculation reduction, which precede neurodegeneration and cognitive defects in aged brain [50–52]. Further, the AD-like neurodegeneration pathogenic cascade is accelerated in pericyte-deficient mice [11]. These point to a critical role of pericyte-mediated vascular integrity in the protection against the occurrence of neurodegenerative diseases, including AD. In the study, we found that miR-181a overexpression was negatively associated with pericyte loss and BBB breakdown in APP/PS1 mice, as shown by immunofluorescent analysis and decreased vascular leakage of IgG. Microvascular reduction has been documented in pericyte-deficient mice [50]. We doubt that miR-181a may also rescue microvascular reduction in APP/PS1 mice, however, whether this is the case needs further evidence. In any rate, these observations associate the protected pericytes, maybe not proven causally at present, with miR-181a alleviating effect on AD progression in APP/PS1 mice. On the other side, we suppose it’s impossible that pericyte is the sole cellular target of miR-181a in CNS. Further studies are required to elucidate whether other cell types, such as astrocytes, microglia, neurons, endothelial cells and even local immune cells [9], contribute to miR-181a function in AD.

Under the treatment of TNF-α and an advanced glycation endproduct, the activation of transcriptional factor FOXO1 can induce pericyte apoptosis [30, 53]. The inhibition of FOXO1 also reduces pericyte apoptosis in Zucker diabetic fatty rats [54]. Consistent with the promotive role of FOXO1 in pericyte apoptosis, in an *in vitro* system imitating Aβ accumulation, we demonstrate that miR-181a protects against pericyte apoptosis by directly targeting FOXO1. Another study has reported that inhibition of miR-181a promotes apoptosis of cervical cancer cells through PTEN/Akt/FOXO1 [55]. It is interesting to test whether the PTEN/Akt is also the upstream signaling factors that dictates miR-181a role in suppressing pericyte apoptosis.

In summary, we provide several lines of evidence which may identify miR-181a as a novel miRNA regulator of AD progression, and also associate its function with protection for pericytes and BBB integrity, thus providing an another example highlighting the importance of antagonizing pericyte lose in modifying the disease progression of AD.

## MATERIALS AND METHODS

### Animals

**Figure 8:**
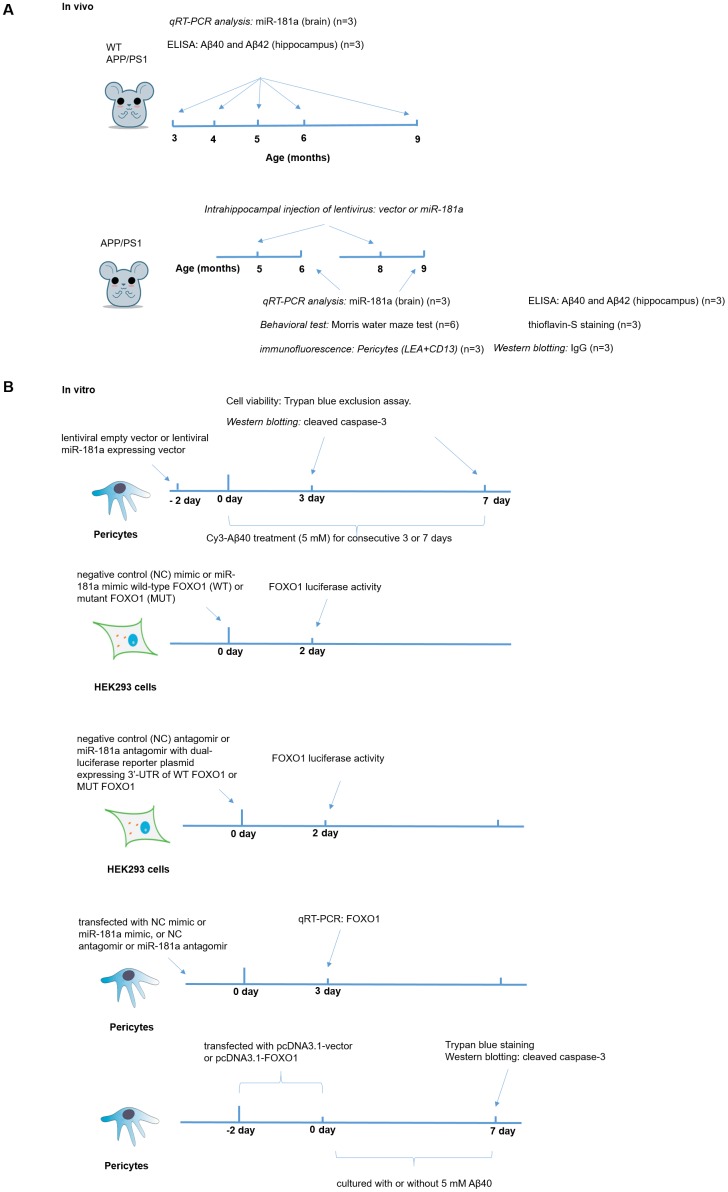
**The experimental procedures *in vivo* and *in vitro*.** (**A**) The experimental procedures *in vivo*, which included ELISA, qRT-PCR, Measurement of Aβ40 and Aβ42, Intrahippocampal injection of lentivirus, immunofluorescence and Behavioral test. (**B**) The experimental procedures *in vitro*, which included Culture and treatment of murine brain pericytes and Western blotting.

The male wild-type C67BL/6J mice (WT) and APP/PS1 transgenic mice were purchased from the Institute of Laboratory Animal Science, Chinese Academy of Medical Sciences (License No. SCXK2014-0004). All animals were allowed free access to food and water and maintained under a facility with 12-h light/dark cycle and constant temperature. All animal experimental procedures were conducted in accordance with the approved protocols enacted by the animal ethics committee of Institute of Microcirculation, Chinese Academy Medical Sciences & Pecking Union Medical College. The experimental procedures *in vivo* could be found in Figure 8A.

### qRT-PCR analysis

Three mice in each group with increasing age were anesthetized with sodium pentobarbital and brain tissues were removed and homogenized in TRIzol reagent (15596018, ThermoFisher Scientific) on ice for extracting total RNA, from which cellular miRNAs were enriched using a miRNeasy Micro Kit (217084, Qiagen). MiRNAs were then reversely-transcribed into complementary cDNA through a Universal cDNA Synthesis Kit II (203301, Exiqon) according to the manufacturer’s protocols. For quantifying miR-181a level, qRT-PCR analysis was performed using a SYBR Premix Ex Taq II kit (Cat. #RR820A, TaKaRa) on a 7500 Real-Time PCR Systems (Applied Biosystems). The sequences of utilized primers are retrievable upon request. The expression of U6 was used as an internal control to normalize data. Results were analyzed with a comparative (Ct) critical threshold, and the relative expression was calculated based on the formula 2^-ΔΔCt^ [56].

### Measurement of Aβ40 and Aβ42

The concentration of Aβ40 and Aβ42 in the hippocampus of WT and APP/PS1 mice was measured through an enzyme-linked immunosorbent assay (ELISA) as described in a previous study [57]. In brief, mice were anesthetized and brain tissues were removed and homogenized RIPA buffer (89900, ThermoFisher Scientific) on ice, and then centrifuged at 12,000 rpm for 10 min to obtain protein samples. Protein concentration was quantified by BCA assay and equal amount of proteins were transferred to a 96-well plate. Each sample was tested in 3 replicates. The concentration of Aβ40 and Aβ42 was measured using a Amyloid beta 40 Mouse ELISA Kit (Cat #KMB3481, Invitrogen) and a Amyloid beta 42 Mouse ELISA Kit (Cat # KMB3441, Invitrogen) according to the manufacturer’s instructions. Results are presented as ng Aβ 40 or Aβ42 per mg total protein.

### Intrahippocampal injection of lentivirus

Lentiviral vector pEZX-MR03 (LV-vector) and that carrying the overexpressing fragment of mouse miR-181a (LV-miR-181a) were purchased from Genecopoeia (Rockville, MD, USA). Lentiviral particles were produced by transfecting 2 μg plasmids of LV-vector or LV-miR-181a into 293T cells per well using Lipofectamine 2000 (11668019, ThermoFisher Scientific). Lentiviral particles (1.0×10^9^ titer) were injected into the hippocampus of APP/PS1 mice aged 5-month-old or 8-month-old as previously described [58]. Each group includes 6 mice. Briefly, mice were anesthetized with pentobarbital sodium and fixed in a stereotaxic apparatus. Then, 2 μl volume of lentiviral particles of LV-vector or LV-miR-181a were injected bilaterally into the hippocampus using a Hamilton 5 μl syringe and a 27-gauge needle at 0.4 μl/min for 5 min duration.

### Behavioral test

The spatial memory performance of mice was assessed by the Morris water maze test [59]. Briefly, in black-painted circular water tank containing water (24 °C), mice were allowed to find a platform within 60 s, which was hidden 1 cm below water surface at the center of target quadrant. Mice unable to find the platform were manually guided to the site of platform. This trail was performed 4 time in each day. In each trial, mice were placed in water at randomly selected 1 of 4 starting position. The elapsed time for finding the platform was defined as the escape latency for each trial. As described above, the post-training probe trial test was conducted for 5 days after training without the platform. In addition, mice were allowed to swim for 60 s at the starting point opposite to the target quadrant, and the number of platform crossing and duration in the target quadrant were recorded.

### Tissue preparation and immunofluorescence

Mice were anesthetized with pentobarbital sodium and transcardially perfused with PBS containing 5 U/ml heparin. Brains were dissected, cryosectioned with a thickness of 14–18 mm, and fixed in ice-cold acetone. Sections were blocked with 5% normal goat serum for 1 h at room temperature and incubated overnight with primary antibody goat anti-CD13 (R&D Systems; AF2335; 1:200) which was diluted in blocking solution at 4 °C. Sections were washed in PBS and incubated with the secondary antibody Alexa 568-conjugated donkey anti-goat (Invitrogen; A11057; 1:200). Further, sections were stained with Dylight 488-conjugated tomato lectin (DL-1174, 1:100, Vector Laboratories) and coverslipped with fluorescent mounting medium (Dako, Carpinteria, CA, USA) to visualize brain microvessel with a 510 confocal microscopy (Zeiss). For thioflavin-S staining, brain sections were stained for 10 min with 0.2% thioflavin-S (T1892, Sigma-Aldrich) diluted in PBS. After repeated wash with PBS, brain sections were imaged using an IX53 fluorescence microscope (Olympus). The quantification of images was analyzed using the Image J software.

### Culture and treatment of murine brain pericytes

The murine brain pericytes were isolated from the microvessel fragments of mouse cortex and hippocampus referenced our previous method [60]. The isolated microvessels were cultured in Pericyte Medium (Catalog Number: 1201, ScienCell), which consists of 500 ml of basal medium, 10 ml of fetal bovine serum, 5 ml of pericyte growth supplement, and 5 ml of penicillin/streptomycin solution. The non-adherent microvessels were removed after 48 h. Pericytes were passaged every 5 days. For Amyloid-beta Protein (1-40) - Cy3 Labeled (Cy3-Aβ40) (FC3-018-01, Phoenix Pharmaceuticals) treatment, pericytes were cultured for consecutive 3 or 7 days in the presence and absence of 5 mM Cy3-Aβ40. Fresh medium with and without 5 mM Cy3-Aβ40 was replaced every 2 days until the end of the experiment. Cell viability was evaluated using Trypan blue exclusion assay. The experimental procedures *in vitro* could be found in Figure 8B.

### Western blotting

Tissue and cellular samples were lysed in RIPA buffer (89900, ThermoFisher Scientific) supplemented with protease inhibitor cocktail (P8340, Sigma-Aldrich), and then protein samples were separated by SDS-PAGE gel and transferred to nitrocellulose membranes (LC2001, ThermoFisher Scientific). Membranes were blocked with 5% milk, and sequentially incubated with primary antibodies at 4 °C and HRP-conjugated secondary antibodies at room temperature. Antibodies were listed as follows: anti-IgG (ab190475, Abcam), anti-β-Actin (ab179467, 1:2000, Abcam), anti-cleaved caspase-3 (#9661, 1:1000, Cell Signaling), anti-FOXO1 (#2880, 1:1000, Cell Signaling), and HRP-conjugated goat anti-mouse IgG (62-6520, ThermoFisher Scientific) and HRP-conjugated goat anti-rabbit IgG (G-21234, ThermoFisher Scientific). After repeated rinse, membranes were incubated with Western ECL detection substrates (32209, ThermoFisher Scientific) to develop protein blots. The analysis of protein blots was performed using the Image J software.

### Data statistics

All data are expressed as means ± SD, and statistical significance was calculated using one-way ANOVA followed by Tukey’s post-hoc test with the SPSS 23.0 (IBM SPSS, Chicago, IL, USA). P < 0.05 was considered statistically significant.
